# Painless extensive ossification of the Achilles tendon: a diagnostic trap?

**DOI:** 10.11604/pamj.2014.17.120.3935

**Published:** 2014-02-18

**Authors:** Ali Akhaddar

**Affiliations:** 1 Department of Neurosurgery, Avicenne Military Hospital, Marrakech, Morocco; 2 University of Mohammed V Souissi, Rabat, Morocco

**Keywords:** Achilles tendon, ossification, trauma

## Image in medicine

A 52-year-old man, previously healthy with no known metabolic or systemic illness, presented acutely following a direct trauma of the right foot. On examination there was soft tissue swelling and tenderness around the dorsum of the foot without neurological deficit. Plain radiography of the foot and the leg revealed a 10 centimeters ossification within the right Achilles tendon without fracture. The patient had no previous ankle problems. Local examination revealed a painless palpable gap and hard edges in the Achilles tendon but there are no disorders in walking. Because the patient was asymptomatic, no surgery was performed. Ossification of the Achilles tendon is an unusual clinical condition to be distinguished from the more frequently occurring tendon calcification. It is characterized by the presence of one or more segments of variable sized ossified mass within the fibrocartilaginous substance of the Achilles tendon. The etiology of this local ossification is unknown. The major contributing factors are trauma (especially repetitive microtrauma) and surgery with other minor causes such as systemic diseases, metabolic conditions, and infections. A large and extensive ossification for more than half of the tendon is rare and should not be misdiagnosed as a fracture or a foreign body particularly following an injury.

**Figure 1:**
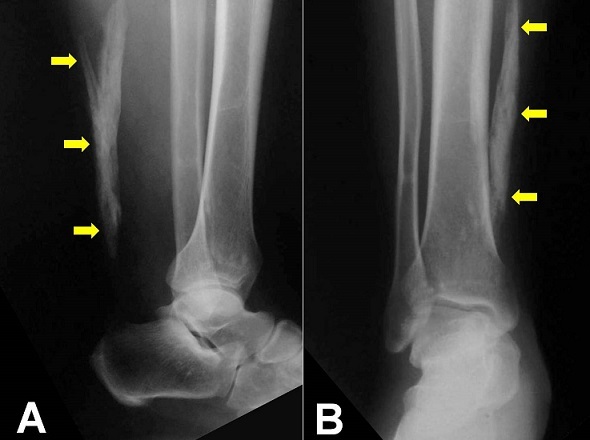
Plain radiographs of the right leg and ankle. Lateral view (A) and antero-posterior (B) view revealing a 10 centimeters ossification within the right Achilles tendon without fracture

